# Evidence for improved memory from 5 minutes of immediate, post-encoding exercise among women

**DOI:** 10.1186/s41235-017-0068-1

**Published:** 2017-08-23

**Authors:** Steven B. Most, Briana L. Kennedy, Edgar A. Petras

**Affiliations:** 1 School of Psychology, The University of New South Wales, Sydney, NSW 2052 Australia; 2 University of Southern California, Los Angeles, USA; 3 Indiana University School of Medicine, Indianapolis, USA

**Keywords:** Retrograde memory enhancement, Memory consolidation, Exercise, Stress hormones, Paired associations, Sex differences

## Abstract

Memories consolidate over time, with one consequence being that what we experience *after* learning can influence what we remember. In these experiments, women who engaged in 5 minutes of low-impact exercise immediately after learning showed better recall for paired associations than did women who engaged in a non-exercise control activity. In experiments 1 and 2, this benefit was apparent in a direct comparison between exercise and non-exercise groups. In experiment 3, it was reflected in a weak, positive correlation between memory performance and exercise-induced change in heart rate. In experiment 4, similar patterns emerged, although they fell short of statistical significance. Such memorial benefits did not emerge among male participants. In experiment 1, half the participants alternatively engaged in an equivalent period of exercise prior to learning, with no benefits for retention of the learned material, suggesting that the memorial benefits of exercise-induced arousal may reflect a specific impact on post-learning processes such as memory consolidation. A meta-analysis across the experiments revealed a reliable benefit of post-learning exercise among women. Variation in the strength of the effect between experiments is consistent with a literature suggesting small but reliable benefits of acute exercise on cognitive performance.

## Significance

Cognitive benefits of chronic exercise have been widely reported; but recently accumulating evidence suggests that even acute bouts of exercise—even after encoding—can lead to measurable improvements in memory. Such a possibility holds practical, in addition to theoretical, implications, as exercise routines can easily be introduced into settings where memory and learning are of central concern (e.g., nursing homes and schools). As of yet, the parameters underlying such putative benefits are little understood: for example, how much exercise is optimal, how long before or after encoding, and for whom are such benefits most apparent? Although some recent evidence suggests that post-encoding exercise enhances memory only with a several hour delay between encoding and exercise, the present results provide evidence that post-encoding exercise can enhance memory when engaged in immediately after learning and for as little as 5 minutes.

## Background

One potentially adaptive consequence of the relatively slow time-course of memory consolidation may be that it allows subsequent processes to modulate the strength of a memory trace (Dudai, 2012; McGaugh, 2000; Redondo & Morris, 2011; Squire, Genzel, Wixted, & Morris, 2015). For example, strong arousal often signifies the importance of an event or stimulus, and the arousal triggered by an experience has been linked to enhanced memory of that experience (Dolan, 2002; McGaugh, 2006; Sharot, Martorella, Delgado, & Phelps, 2007).

Memorial benefits also extend to material learned just prior to an arousal induction (e.g., Anderson, Wais, & Gabrieli 2006; Knight & Mather, 2009; McGaugh, 2006; Nielson & Powless, 2007; Nielson, Yee, & Erickson, 2005; Segal, Cotman, & Cahill, 2012). In one study, students who watched an emotionally arousing film clip after a classroom lecture performed better on a test on the material 2 weeks later than did students who watched an emotionally neutral clip (Nielson & Arentsen, 2012).

Research from both the human and animal literatures converge in support of the notion that stress hormones play a role in solidifying memory of material learned just prior to the arousal induction. For example, when rats were administered stress hormones after a training phase, they subsequently exhibited enhanced memory for their training; however, when the stress hormones were blocked by beta-adrenergic antagonists, their behavior indicated impaired memory compared to counterparts who received stress hormones without beta-adrenergic antagonists (Liang, Juler, & McGaugh, 1986). In another study, rats who had access to a running wheel immediately after extinction training (following fear conditioning) exhibited lower freezing behavior when re-exposed to the fear-conditioned context (Siette, Reichelt, & Westbrook, 2014). Similarly, when human participants were intravenously administered doses of epinephrine immediately after viewing a series of pictures, they freely recalled more items a week later than did those participants who were injected with saline instead (Cahill & Alkire, 2003). Because the administration of stress hormones occurred after learning, their impact could not be attributed to changes in motivation, emotion, or attention during encoding. Notably, the impact of stress hormones on memory consolidation is not uniform across all individuals: a growing number of studies have documented sex differences in the modulatory impact of stress on memory (e.g., Andreano, Arjomandi, & Cahill, 2008; Andreano & Cahill, 2006; Felmingham, Fong, & Bryant, 2012; Jackson et al., 2005; Nielsen, Segal, Worden, Yim, & Cahill, 2013; Wolf et al, 2001).

The impact of post-encoding arousal seems to generalize to arousal induced by exercise, consistent with findings that exercise can facilitate the release of stress hormones in both rats and humans (Chatterton, Vogelsong, Lu, Ellman, & Hudgens, 1996; Pagliari & Peyrin, 1995). This carries practical implications in addition to theoretical ones, given the relative feasibility of introducing exercise into contexts where pharmacological and emotional manipulations would be less desirable (e.g., schools, nursing homes, etc.). Indeed, when older adults exercised on a bicycle for 6 minutes after rating images, they recalled more of the images in a surprise memory test the next day (Segal et al., 2012). Similarly, Nielson and colleagues had participants read highlighted words in paragraphs while they sometimes tightened their muscles using a hand dynamometer (Nielson & Jensen, 1994; Nielson, Radtke, & Jensen, 1996). When participants tightened their arm muscles during and after the learning phase, delayed recall and delayed recognition memory for the words were enhanced compared to when they did not exert physical effort. This enhancement was especially pronounced when the manipulation took place during the consolidation or retrieval phase of memory (Nielson et al., 1996), but it was not observed in subjects who were taking beta-blockers (Nielson & Jensen, 1994). Healthy young participants also showed better retention of motor skills when learning was followed by 20 minutes of intense cycling (Roig, Skriver, Lundbye-Jensen, Kiens, & Nielsen, 2012). Such findings fit in well with a broader literature on the cognitive impact of a single session of exercise (i.e., “acute exercise”), which has tended to find small but reliable benefits (e.g., Chang, Labban, Gapin, & Etnier, 2012).

In one recent study, evidence suggested that the retrograde benefits of exercise emerged only when a sufficient delay separated encoding and physical exertion. Participants learned picture–location associations and then rested or performed 35 minutes of physical exercise either immediately afterwards or 4 h afterwards (Van Dongen, Kersten, Wagner, Morris, & Fernández, 2016). Those who exercised 4 h after encoding exhibited enhanced memory relative to non-exercisers, whereas those who exercised immediately afterwards did not. The fact that the memorial benefits from delayed exercise were not also observed in the immediate exercise condition seemed to run counter to predictions of well-established consolidation theories (e.g., Redondo & Morris, 2011), and it was hypothesized that the neural processes immediately after encoding were already optimal for learning such that they could not be further enhanced via exercise. Given the paucity of current knowledge about the time-course of and conditions modulating consolidation in humans, the researchers encouraged interpretive caution pending further investigation.

The present study investigated whether 5 minutes of acute exercise immediately after learning can enhance memory for paired associations among healthy young adults, taking into account such potential factors as sex differences, which have been found to impact stress-modulated memory consolidation (Andreano & Cahill, 2006; Zorawski, Blanding, Kuhn, & LaBar, 2006). The short duration of exercise in the experiments is of interest, as exercise-induced changes in the levels of different stress hormones appear to follow different time courses (e.g., with changes in adrenalin and noradrenalin occurring early during moderate exercise and ACTH and cortisol changes occurring later; De Vries, Bernards, De Rooij, & Koppeschaar, 2000). Thus, effects on memory from 5 minutes of exercise might provide important information in trying to pinpoint the roles of particular stress hormones on memory consolidation.

## Experiment 1: Exercise-induced, retrograde memory enhancement for names and faces

### Methods

#### Participants

Eighty-two introductory psychology undergraduate students (mean age 19.9 years) participated, mid-second semester, for course credit. Data from eight were excluded from analyses due to failure to follow directions or incomplete data, leaving 38 females and 36 males in the study. All participants provided informed consent, as approved by the University of Delaware Human Subjects Review Board, and indicated that they had no known medical conditions that would prevent them from engaging in a low-impact cardio exercise.

#### Materials and procedure

Following from previous research showing recruitment of a range of neural regions when learning and remembering name–face pairs (Zeineh, Engel, Thompson, & Bookheimer, 2003), stimuli were paired faces and names. Name–face pairs were presented on a CRT monitor with an 800 × 600 resolution using Psychophysics Toolbox for Matlab (Brainard 1997; Pelli 1997). Face pictures were 15 full-color 258 × 350 pixel photographs of men with neutral expressions, collected from the Karolinska Directed Emotional Faces database (Lundqvist, Flykt, & Öhman 1998). Names were common male names in the United States (Joseph, David, William, Richard, Michael, Robert, John, Thomas, James, Charles, Daniel, Paul, Mark, Donald, and Christopher) and were presented in black, Times New Roman, 120 point font. Each face appeared in the center of the screen above its associated name.

The learning and testing phases were conducted 24 h apart from each other. Thirty-eight participants were assigned to the post-learning activity procedure (ten females and nine males in both the exercise and the non-exercise conditions), and 36 participants were assigned to the pre-learning activity procedure (nine females and nine males in both the exercise and the non-exercise conditions). The groups were run sequentially, starting with the post-learning activity procedure.

##### Post-learning activity procedure

During the learning phase (day 1), participants sat at a comfortable distance from a computer screen and were told that they would later be tested on their memory for name–face pairs that would appear on the screen. Each name–face pair appeared once for 4 s in each of seven blocks. Presentation order within each block was random, with the constraint that the same pair was not repeated twice in a row (i.e., at the end of one block and the start of the next).

Participants were also asked to rate their emotional state in order to isolate the effects of physical arousal from those of emotional arousal: if emotional arousal were to differ between the exercise and non-exercise groups, the intention was to assess the impact of exercise while controlling for emotional arousal. Immediately after the learning period, participants rated their subjective emotional state in terms of valence (positive versus negative) and arousal (high versus low) by placing a mark within a 9 × 9 “affect grid” (Russell, Weiss, & Mendelsohn, 1989), wherein the horizontal extent represented valence and the vertical extent represented arousal. Baseline heart rate (beats per minute; bpm) was measured via a fingertip pulse oximeter (CMS-50DL). Participants then engaged in one of two post-learning activities. The learning phase and the post-learning activity were separated by approximately 1 minute.

Participants engaged either in 5 minutes of step-exercise (exercise condition) or 5 minutes of quiet activity, during which they built freely with connecting toy pieces (*K’Nex*; non-exercise condition). The exercise condition involved repeatedly stepping on and off of a 6.5” step, self paced, while counting the number of steps made. The non-exercise condition involved freely joining pieces together while keeping count of the number of pieces used. Counting was used in both conditions as a means to discourage participants from rehearsing information from the learning phase. An experimenter used a stopwatch to time both the exercise and non-exercise activities and told the participant to stop at the end of 5 minutes. The two types of post-learning activity were performed alone in a small room, behind a closed door, and only one participant was run at a time. After engaging in the activity, subjective emotional state and heart rate were measured again via an affect grid and fingertip pulse oximeter.

Twenty-four hours later, participants returned to complete a memory test (day 2). Each participant received a worksheet with the 15 faces from the previous day’s learning phase and was instructed to write the corresponding name beneath each picture. This test was untimed, and participants were encouraged to guess when they were unable to remember a name but could leave an answer blank if they were unable to guess. Participants were then debriefed.

##### Pre-learning activity procedure

The materials and procedure were the same as in the post-learning activity procedure, with the exception that the exercise versus non-exercise manipulation was administered prior to learning. Participants first provided baseline measures of heart rate and self-report valence and arousal levels, and immediately engaged in their assigned pre-learning activity. Post-activity measures of heart rate and affect were recorded, and participants then viewed the names and faces in the learning portion of the experiment. After the learning phase, heart rate and affect measures were taken and participants performed paper-and-pencil mazes for 5 minutes as a means to equate immediately subsequent activity between groups. Twenty-four hours later, participants returned to the lab to complete the memory test.

#### Data analysis

Answers were coded as correct when participants recalled the exact name, substituted a common nickname (e.g., “Mike” for “Michael”), or had an obvious misspelling (e.g., “Michal”). All other responses were considered incorrect. Every participant therefore had a score based on number of correct responses, with 0 representing none correct and 15 representing perfect memory performance.

Performance was analyzed using the raw number of correct responses. Mood valence and arousal measures were based on independent 9-point scales (with 1 corresponding with negative emotional valence/low arousal and 9 corresponding with positive emotional valence/high arousal). Percent change in heart rate was calculated by subtracting pre-activity from post-activity heart rate and dividing by pre-activity heart rate, in order to accommodate expected variation in baseline heart rate (e.g., an increase of 10 bpm might have different implications following a baseline heart rate of 60 bpm compared to a baseline of 110 bpm). Change in affect measures were calculated by simply subtracting post-activity measures minus pre-activity measures, as assumptions about the relative meaning of (for example) an increase of 2 units of valence rating may be unwarranted, given the inherently subjective nature of this measure.

### Results

A 2 (activity timing: pre- versus post-learning) × 2 (activity type: exercise versus non-exercise) × sex (male versus female) ANOVA on number of correct responses revealed no main effects of activity timing (*F*(1, 66) = 0.81, *p* = 0.372, *η* ^2^ = 0.009), activity type (*F*(1, 66) = 0.80, *p* = 0.375, *η* ^2^ = 0.010), or sex (*F*(1, 66) = 0.03, *p* = 0.856, *η* ^2^ < 0.001). No two-way interactions with sex emerged (*ps* > 0.34). However, a significant activity timing × activity type interaction emerged (*F*(1, 66) = 7.38, *p* = 0.008, *η* ^2^ = 0.091), as did the three-way interaction between activity timing, activity type, and sex (*F*(1, 66) = 4.00, *p* = 0.05, *η* ^2^ = 0.049). These are explored in more detail below, considering first the impact of exercise (versus non-exercise) after learning and then the impact of exercise (versus non-exercise) before learning. In the exercise condition, there was no difference in the number of steps reported taken by men and women (men, mean (M) = 121.7, standard deviation (SD) = 53.8; women, M = 138.3, SD = 51.1; *t*(33) = 0.93, *p* = 0.36; data missing from two participants). That said, number of steps constituted the only measure in the study that was both non-subjective and non-verifiable, and—as the intended purpose of the counting task was simply to discourage rehearsal of the learned material—participants’ instructions did not emphasize counting accuracy. Thus, we do not include this factor in our subsequent analyses. (An improvement in future follow-up studies may be to track number of steps more objectively).

#### Post-learning activity procedure

**Fig. 1:**
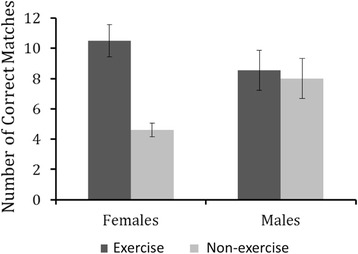
Means (and standard errors) of correct items reported in the memory test in the post-learning activity procedure from experiment 1

Participants who performed exercise after the learning phase exhibited significantly better memory for name–face pairs 1 day later (M = 9.58, SD = 3.72) than those who engaged in the non-exercise activity after learning (M = 6.37, SD = 3.37; *t*(36) = 2.789, *p* = 0.008, *d* = 0.90) (Fig. 1).

##### Males *versus* females

A 2 (activity: exercise versus non-exercise) × 2 (sex: female versus male) ANOVA revealed a significant main effect of activity (*F*(1, 34) = 7.831, *p* = 0.008, *η* ^2^ = 0.16), no significant main effect of sex (*F*(1, 34) = 0.276, *p* = 0.603, *η* ^2^ = 0.006), and a significant interaction between them (*F*(1, 34) = 5.259, *p* = 0.028, *η* ^2^ = 0.11) (Fig. 1).

Women in the exercise group remembered more than women in the non-exercise group (M = 10.5, SD = 3.41 versus M = 4.9, SD = 1.97, t(18) < 0.001, *d* = 2.01). However, men in the exercise group (M = 8.56, SD = 3.97) performed no differently to men in the non-exercise group (M = 8.0, SD = 3.94) (t(16) = 0.298, *p* = 0.77, *d* = 0.14). Women performed worse than men in the non-exercise condition (t(17) = 2.21, *p* = 0.04, *d* = 0.995), but not in the exercise condition (t(17) = 1.15, *p* = 0.27, *d* = 0.52).

##### Mood measures

Collapsed across men and women, no differences in self-reported mood measures of valence emerged (exercise, M = +0.58, SD = 1.87; non-exercise, M = +0.05, SD = 1.84; *t*(36) = 0.876, *p* = 0.39), nor of arousal (exercise, M = + 0.95, SD = 2.84; non-exercise, M = + 1.05, SD = 1.75; *t*(36) = −0.138, *p* = 0.89). There were no between-activity differences in self-reported mood measures of valence or arousal in either women or men (*ts* < 1.40, *ps >* 0.18).

##### Heart rate

Heart rate increased to a greater degree in the exercise condition than in the non-exercise condition when collapsed across participants (*t*(36) = 3.41, *p* = 0.002, *d* = 1.17), when limited only to the women (*t*(18) = 2.89, *p* = 0.010, *d* = 1.28), and marginally when limited only to the men (*t*(16) = 3.41, *p* = 0.070, *d* = 0.97). Following exercise, men and women did not differ in their change of heart rate (*t*(17) = 1.12, *p* = 0.28, *d* = 0.48). Among women, collapsed across activity, increase in heart rate correlated with memory accuracy. Means and standard deviations of heart rate change are reported in Table 1, along with the correlations of heart rate change with memory performance.

**Table 1:** Means (and standard deviations) of percentage heart rate increase following exercise and non-exercise activities, as well as bivariate correlations between percentage heart rate increase and memory performance, for women, men, and combined

	Women	Men	Combined
Experiment 1			
Post-learning activity			
Exercise			
Mean HR change	32% (22%)	21% (24%)	27% (23%)
Correlation with memory	*r(8)* = 0.05	*r(7)* = −0.10	*r(17)* = 0.04
	*p* = 0.88	*p* = 0.80	*p* = 0.86
Non-exercise			
Mean HR change	9% (14%)	4% (11%)	6% (13%)
Correlation with memory	*r(8)* = 0.09	*r(7)* = 0.01	*r(17)* = −0.07
	*p* = 0.82	*p* = 0.98	*p* = 0.79
Collapsed across activity			
Mean HR change	21% (21%)	12% (20%)	16% (21%)
Correlation with memory	*r(18)* = 0.44	*r(16)* = −0.02	*r(36)* = 0.21
	*p* = 0.05	*p* = 0.93	*p* = 0.20
Pre-learning activity			
Exercise			
Mean HR change	40% (18%)	25% (18%)	32% (19%)
Correlation with memory	*r(7)* = −0.29	*r(7)* = 0.28	*r(16)* = 0.03
	*p* = 0.46	*p* = 0.47	*p* = 0.92
Non-exercise			
Mean HR change	9% (14%)	5% (11%)	7% (12%)
Correlation with memory	*r(7)* = −0.13	*r(7)* = −0.41	*r(16)* = −0.19
	*p* = 0.74	*p* = 0.28	*p* = 0.44
Collapsed across activity			
Mean HR change	24% (22%)	15% (18%)	20% (20%)
Correlation with memory	*r(16)* = −0.36	*r(16)* = 0.001	*r(34)* = −0.17
	*p* = 0.14	*p* > 0.99	*p* = 0.32
Experiment 3			
Exercise			
Mean HR change	51% (30%)	52% (27%)	52% (29%)
Correlation with memory	*r(33)* = 0.29	*r(22)* = −0.01	*r(57)* = 0.19
	*p* = 0.096	*p* = 0.96	*p* = 0.16
Non-exercise			
Mean HR change	10% (22%)	11% (16%)	10% (20%)
Correlation with memory	*r(35)* = 0.19	*r(18)* = −0.43	*r(55)* = −0.01
	*p* = 0.25	*p* = 0.057	*p* = 0.96
Experiment 4			
Exercise			
Mean HR change	44% (22%)	24% (30%)	40% (25%)
Correlation with memory	*r(27)* = 0.03	*r(6)* = −0.48	*r(35)* = −0.02
	*p* = 0.86	*p* = 0.23	*p* = 0.92
Non-exercise			
Mean HR change	5% (12%)	7% (19%)	6% (14%)
Correlation with memory	*r(27)* = 0.17	*r(7)* = 0.14	*r(36)* = 0.16
	*p* = 0.38	*p* = 0.71	*p* = 0.35
Collapsed across activity			
Mean HR change	25% (26%)	15% (26%)	22% (26%)
Correlation with memory	*r(56)* = 0.16	*r(15)* = −0.17	*r(73)* = 0.10
	*p* = 0.22	*p* = 0.51	*p* = 0.38

Correlation statistics did not substantively change when mood self-reports of change in valence and arousal were partialled out. Experiment 3 involved repeated measures, rendering observations in the exercise and non-exercise conditions non-independent of each other; thus, we do not collapse across activity for this experiment. *HR* heart rate

#### Pre-learning activity procedure

In contrast to the results from the post-learning activity procedure, in the pre-learning activity procedure those in the exercise group (M = 6.44, SD = 3.91) performed somewhat worse than those in the non-exercise group (M = 8.00, SD = 3.83) on the memory task, although this difference was not significant (*t*(34) = 1.20, *p* = 0.24, *d* = 0.40).

##### Males *versus* females

A 2 (activity type) × 2 (sex) ANOVA for memory scores revealed no significant main effect of activity (*F* = 1.404, *p* = 0.245, *η* ^2^ = 0.04), no main effect of sex (*F* = 0.459, *p* = 0.503, *η* ^2^ = 0.013), and no interaction between them (*F* = 0.459, *p* = 0.503, *η* ^2^ = 0.013). In the subsequent planned comparisons, women in the exercise condition (M *=* 6.44, SD *=* 3.64) showed a pattern suggesting worse performance than those in the non-exercise condition (M *=* 8.89, SD *=* 4.23), although this did not reach statistical significance (*t*(16) = 1.314, *p* = 0.21, *d* = 0.62). A similar non-significant pattern emerged among men (exercise, M *=* 6.44, SD *=* 4.39; non-exercise, M *=* 7.11, SD *=* 3.41; *t*(16) = 0.360, *p* = 0.72, *d* = 0.17).

##### Mood measures

Collapsed across men and women, there was no main effect of pre-learning activity on self-reported ratings of change in emotional arousal (exercise, M = +1.18, SD = 1.67; non-exercise, M = +0.50, SD = 1.58; *t*(33) = 0.1.232, *p* = 0.23), and the difference in self-reported mood valence was only marginal (exercise, M = −0.06, SD = 1.43; non-exercise, M = +1.00, SD = 1.78; *t*(33) = 1.929, *p* = 0.062).^1^ No differences in arousal or valence emerged among the men or women alone (*t*s < 1.95, *p*s ≥ 0.07).

##### Heart rate

Heart rate increased to a greater degree in the exercise condition than in the non-exercise condition when collapsed across participants (*t*(34) = 4.68, *p* < 0.001, *d* = 1.61), when limited only to the women (*t*(16) = 4.05, *p* = 0.001, *d* = 1.94), and when limited only to the men (*t*(16) = 2.74, *p* = 0.015, *d* = 1.38). Following exercise, men and women did not differ in their change of heart rate (*t*(16) = 1.73, *p* = 0.10, *d* = 0.83). Means and standard deviations of heart rate change are reported in Table 1, along with the correlations of heart rate change with memory performance.

### Discussion

Five minutes of post-learning exercise appeared to enhance memory for paired associations among women, but an equivalent period of exercise prior to learning yielded no similar benefit. Memorial benefits of acute exercise may stem primarily from its impact on post-learning processes such as consolidation. The exercise-induced benefit in the post-learning activity procedure appeared to be partly driven by women’s lower accuracy (relative to men’s) in the control condition (Fig. 1). Experiment 2, which aimed to replicate and extend the post-learning activity findings, provided an additional opportunity to assess whether this pattern was spurious.

## Experiment 2: Short-interval, retrograde enhancement of learned associations

In experiment 2, we sought to replicate and extend the post-learning findings of experiment 1, and it differed from experiment 1 in the following ways. First, memory was tested on the same day as learning in order to assess whether the retrograde benefits of exercise could be observed after a shorter interval and without sleep consolidation as a possible influence (Stickgold, 2005). Second, a different non-exercise activity was employed in order to control for the possibility that the apparent benefits of exercise in experiment 1 might actually have stemmed from interference from the visuospatial nature of the control task. Indeed, previous work has shown that activities that reduce retroactive interference after learning lead to less forgetting of the learned material (e.g., Cowan, Bechin, & Della Sala, 2004; Jenkins & Dallenbach, 1924), lending credence to the possibility that what appeared to be exercise-induced memory enhancement actually stemmed from retroactive interference from the control task (although the correlation between post-learning heart-rate increase and memory performance in experiment 1 would seem to speak against this alternative).

### Methods

#### Participants

Eighty-three undergraduate introductory psychology students participated, mid-second semester, for course credit (mean age = 19.9 years). Three were excluded from the study: one for incomplete data and two for failure to follow instructions. The remaining data included 41 in the exercise condition (21 females and 20 males) and 39 in the non-exercise condition (19 females and 20 males).

#### Materials and procedure

Materials and procedures were the same as the post-learning activity procedure in experiment 1 with the following exceptions. First, for the non-exercise condition, participants sat and tapped their hands on a table for 5 minutes, tapping their left and right hands in alternation at a pace of their choice while counting the number of taps.

Second, the memory test in this experiment was administered during the same day as the learning phase. Participants in both groups worked on paper-and-pencil mazes for the 10 minutes between the post-learning activity and test phase (to avoid stress due to time pressure, they were informed that it was not necessary to finish the mazes). Third, in the learning phase, participants viewed the name–face pairs four times rather than seven. Heart rate and self-reported mood measures were not recorded.

### Results and discussion

**Fig. 2:**
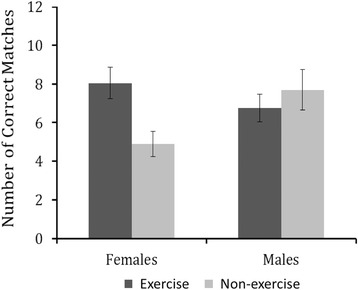
Means (and standard errors) of correct items reported in the memory test in experiment 2

Collapsed across men and women, there was no difference in memory performance between the exercise group (M *=* 7.44, SD *=* 3.54) and non-exercise group (M *=* 6.33, SD *=* 4.13) (*t*(78) = 1.29, *p* = 0.20, *d* = 0.29; Fig. 2). However, when men and women were analyzed separately, the results mirrored those from experiment 1, as described below.

#### Males *versus* females

A 2 (activity) × 2 (sex) ANOVA revealed no main effect of sex (*F*(1,76) = 0.77, *p* = 0.38, *η* ^2^ = 0.009) and no main effect of activity (*F*(1, 76) = 1.82, *p* = 0.18, *η* ^2^ = 0.022), but a significant interaction between them (*F*(1, 76) = 6.21, *p* = 0.015, *η* ^2^ = 0.073) (Fig. 2). Women in the exercise group (M *=* 8.10, SD *=* 3.77) remembered more than women in the non-exercise group (M *=* 4.89, SD *=* 2.865) (*t*(38) = 3.00, *p* = 0.005, *d* = 0.96), but this difference was again not observed among the male participants (exercise, M *=* 6.75, SD *=* 3.23; non-exercise, M *=* 7.70, SD *=* 4.73; *t*(38) = −0.743, *p* = 0.462, *d* = 0.23). Women performed worse than men in the non-exercise condition (*t*(37) = 2.23, *p* = 0.03, *d* = 0.72), but not in the exercise condition (*t*(39) = 1.23, *p* = 0.23, *d* = 0.38). This is consistent with the results of experiment 1, demonstrating a retrograde memory enhancement among women for items that were learned prior to exercise-induced arousal.

## Experiment 3: Generalizing to learned associations between non-social stimuli

In studies of emotion-induced retrograde enhancements of memory, evidence suggests that the benefits are particularly strong for learned material that in itself is emotional (Segal & Cahill, 2009). Although the face stimuli in the preceding experiments wore neutral expressions, faces are inherently social and may have emotional relevance. In experiment 3, we tested whether the retrograde memory benefits of exercise-induced physiological arousal generalized to paired associations with non-social, abstract shapes. In an additional departure from the preceding experiments, the exercise and non-exercise conditions were run as a within-subjects variable to minimize contributions of baseline individual differences to memory performance.

### Methods

#### Participants

Forty-one female and 28 male introductory psychology undergraduate students participated, mid-first semester, for course credit in experiment 3 (69 participants total, mean age = 19.23 years). Twenty-one were excluded from analyses for failure to complete the four-session study described below, leaving 31 women and 17 men in the analyses comparing the exercise to non-exercise conditions. Analyses involving heart rate within conditions included all participants who had completed both days of either the exercise condition (24 men, 35 women) or the non-exercise condition (20 men, 37 women), regardless of whether they had completed both conditions (see below).

#### Materials and procedure

Materials and procedure were the same as the post-learning activity procedure in experiment 1, except for the following. In the first week, participants were randomly assigned to either the exercise or non-exercise condition and viewed 15 shapes paired with names. Shapes were black, abstract images presented on a white background (several of them taken from Fiser & Aslin, 2002). The names were the same as in the preceding experiments; names were used in order to minimize the potential of strategic linking between word meanings and features of the abstract shapes. Regardless of post-learning activity, all participants saw the same 15 shape–name pairs during week 1, with each study item presented five times. In contrast to the preceding experiments, participants were not instructed to count the number of steps or taps made. As in experiment 1, heart rate and affect grid ratings were obtained before and after the post-learning activities. Participants returned to the lab 24 h later to complete a memory test for those shape–name pairs, wherein they wrote the corresponding name underneath each abstract shape. One week after their first visit, they returned for the week 2 session, during which they engaged in the alternative post-learning activity. During week 2, they viewed a new set of 15 shape–name pairs (all participants saw the same 15 shape–name pairs during week 2) and the same testing method was used the following day as in week 1.

### Results and discussion

Overall, memory performance for the shapes and names did not differ between the exercise (M *=* 9.04, SD *=* 3.94), and non-exercise conditions (M *=* 8.83, SD *=* 4.18) (*t*(46) = 0.43, *p* = 0.673, *d* = 0.05).

#### Males versus females

A 2 (activity type) × 2 (sex) mixed ANOVA for memory scores revealed no significant main effect of post-learning activity (*F*(1,46) = 0.01, *p* = 0.92, *η*_*p*_^2^ < 0.001), no main effect of sex (*F*(1,46) = 0.108, *p* = 0.74, *η*_*p*_^2^ = 0.002), and no interaction between them (*F*(1,46) = 1.05, *p* = 0.31, *η*_*p*_^2^ = 0.022).

In contrast to experiments 1 and 2, the planned comparison revealed no significant main effect of post-learning activity in women (exercise, M *=* 9.10, SD *=* 3.92; non-exercise, M *=* 8.52, SD *=* 4.25; *t*(30) = 0.91, *p* = 0.369, *d* = 0.14). Neither was there a main effect of post-learning activity in men (exercise, M *=* 8.94, SD *=* 4.08; non-exercise, M *=* 9.41, SD *=* 4.11; *t*(16) = −0.63, *p* = 0.538, *d* = 0.12).

#### Mood measures

Collapsed across men and women, no between-activity difference in self-reported mood measures of valence emerged (exercise, M = +0.21, SD = 1.38; non-exercise, M = −0.13, SD = 1.59; *t*(47) = 1.55, *p* = 0.14), but those in the exercise condition reported a greater increase in arousal (exercise, M = +2.19, SD = 1.90; non-exercise, M = −0.15, SD = 1.81; *t*(47) = 6.17, *p* < 0.001). There were no between-activity differences in self-reported mood measures of valence in either women or men alone (*ts* < 1.10, *ps >* 28), but both women and men reported a greater increase in arousal following exercise (*ts* > 3.19, *ps* < 0.006). (Including change in self-reported arousal as a covariate in the initial ANOVAs did not substantively change the results).

#### Heart rate

**Fig. 3:**
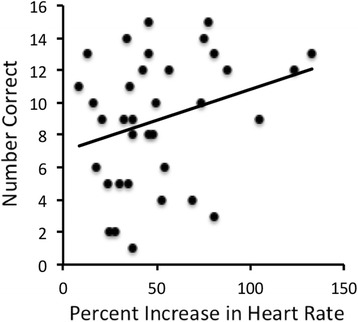
The correlation between exercise-induced change in heart rate and memory performance among women in experiment 3 suggested a positive relationship, although this fell short of statistical significance

Heart rate increased to a greater degree in the exercise condition than in the non-exercise condition when collapsed across participants (*t*(61) = 10.37, *p* < 0.001, *d*_*z*_ = 1.42), when limited only to the women (*t*(36) = 7.08, *p* < 0.001, *d*_*z*_ = 1.21), and when limited only to the men (*t*(24) = 8.45, *p* < 0.001, *d*_*z*_ = 1.97). Men and women did not differ in their change of heart rate following exercise (*t*(60) = 0.61, *p* = 0.95, *d* = 0.03). Among women, there was a trend suggesting a positive correlation between increase in heart rate following exercise and memory accuracy (Fig. 3).^2^ Means and standard deviations of heart rate change are reported in Table 1, along with the correlations of heart rate change with memory performance.

## Experiment 4: Pursuing a replication of experiment 1

A striking aspect of the experiments reported so far is that whereas the memorial impact of post-learning exercise in experiment 1 was very strong, the effect of exercise in experiments 2 and 3 was weaker (though still robust among women in experiment 2). This could be due to adjustments in the experimental designs (e.g., testing immediately after exercise, employing stimuli other than faces, and testing within-subjects). In order to gain a clearer picture of the overall strength of the memorial impact of immediate, post-learning exercise, we conducted a relatively straightforward replication of experiment 1, with the only change being that control participants engaged in tapping as a control activity (as in experiment 2) instead of building with connecting toy pieces. We recruited both female and male participants but prioritized recruitment of female participants due to the more consistent memorial benefits in that group.

### Methods

#### Participants, materials, and procedure

Fifty-eight female and 17 male undergraduate introductory psychology students participated, mid-second semester, for course credit in experiment 4 (mean age = 21.10 years). Materials and procedure were the same as in the post-learning condition in experiment 1 except that participants in the control condition (nine men, 29 women) engaged in the tapping task from experiment 2.

### Results and discussion

Overall, participants who performed exercise after the learning phase exhibited slightly better memory for name–face pairs (M = 7.89, SD = 3.56) than those who engaged in the non-exercise activity after learning (M = 7.13, SD = 3.74), but this difference was not significant (*t*(73) = 0.90, *p* = 0.37, *d* = 0.21).

#### Males versus females

A 2 (activity: exercise versus non-exercise) × 2 (sex: female versus male) ANOVA revealed no significant main effect of activity (*F*(1, 71) = 0.14, *p* = 0.71, *η* ^2^ = 0.002), or of sex (*F*(1, 71) = 1.23, *p* = 0.272, *η* ^2^ = 0.017). Nor was there a significant interaction between them (*F*(1, 71) = 0.421, *p* = 0.519, *η* ^2^ = 0.006).

Examining the data only from the women, those in the exercise group exhibited somewhat better memory than those in the non-exercise group, but this fell short of significance (exercise, M = 8.28, SD = 3.53; non-exercise, M = 7.24, SD = 3.59; *t*(56) = 1.11, *p* = 0.27, *d* = 0.29). Examining the data only from the men, those in the exercise group did not exhibit better memory than those in the non-exercise group (exercise, M = 6.50, SD = 3.51; non-exercise, M = 6.78, SD = 4.41; *t*(15) = 0.14, *p* = 0.89, *d* = 0.07).

#### Mood measures

Collapsed across men and women, self-reported mood measures revealed greater shifts towards positive valence (exercise, M = +0.70, SD = 1.65; non-exercise, M = −0.26, SD = 1.90; *t*(73) = 2.46, *p* = 0.02) and higher arousal (exercise, M = +2.11, SD = 1.71; non-exercise, M = +0.63, SD = 2.15; *t*(73) = 3.51, *p* < 0.001) among those within the exercise condition. Among the women alone, this pattern emerged in ratings of arousal (exercise, M = +2.23, SD = 1.76; non-exercise, M = +0.79, SD = 2.19; *t*(57) = 2.79, *p* = 0.007) but not valence (exercise, M = +0.40, SD = 1.48; non-exercise, M = −0.14, SD = 2.00; *t*(57) = 1.18, *p* = 0.24). Among the men alone, this pattern emerged in ratings of valence (exercise, M = +2.00, SD = 1.83; non-exercise, M = −0.67, SD = 1.58; *t*(14) = 3.13, *p* = 0.007) but not arousal (exercise, M = +1.57, SD = 1.51; non-exercise, M = +0.11, SD = 2.03; *t*(14) = 1.59, *p* = 0.13). (Including self-reported changes in arousal and valence as covariates in the initial ANOVAs did not substantively change the results.)

#### Heart rate

Heart rate increased to a greater degree in the exercise condition than in the non-exercise condition when collapsed across participants (*t*(73) = 7.33, *p* < 0.001, *d* = 1.75) and when limited only to the women (*t*(56) = 8.32, *p* < 0.001, *d* = 2.26), but not when limited only to the men (*t*(15) = 1.39, *p* = 0.18, *d* = 0.68). Following exercise, women exhibited a greater increase in heart rate than men (*t*(35) = 2.13, *p* = 0.04, *d* = 0.78). Among women, collapsed across activity, memory performance exhibited a slight positive relationship with change in heart rate, but this was not significant. Means and standard deviations of heart rate change are reported in Table 1, along with the correlations of heart rate change with memory performance, which were not substantively changed when controlling for changes in mood measures of valence and arousal.

The results of experiment 4 shed light on the strength of the effect under investigation. On one hand, taken alone, the results of this experiment suggest only a weak (i.e., non-significant) impact of immediate post-learning exercise on memory. However, taken together with the findings of experiments 1–3, the results suggest that the impact is strong enough to be measurable. As with the previous experiments, this impact was observed primarily among the female participants: those who exercised immediately after learning exhibited numerically improved memory performance, consistent with the previous experiments. Also consistent with the previous experiments, memory performance among the women showed evidence of a positive relationship with change in heart rate, but this did not approach statistical significance.

## General discussion

Memories consolidate over time, with one consequence being that what we experience *after* learning can influence what we remember (McGaugh, 2006; Redondo & Morris, 2011). For example, in humans, evidence suggests that emotional arousal experienced after learning can facilitate consolidation, leading to increased retention of material, as assessed through recognition and recall tasks (e.g., Anderson et al. 2006; Cahill & Alkire, 2003; Knight & Mather, 2009). The present study built upon recent evidence that exercise-induced arousal after learning serves a similar function (e.g., Segal et al., 2012). In contrast to recent work suggesting that such benefits emerge only with a several hour delay between encoding and exercise (Van Dongen et al., 2016), taken together across four experiments we observed evidence for enhanced memory among women for paired associations when learning was followed immediately by 5 minutes of exercise (experiments 1 and 2) and that the degree of activity-induced physiological arousal—as measured by change in heart rate—predicted memory performance (experiments 1 and 3). This memorial benefit emerged specifically when exercise followed learning, not when it preceded it (experiment 1), supporting the notion that exercise-induced arousal can specifically impact post-learning processes such as consolidation, although it may also be that more variables are necessary to understand precisely why exercise yielded benefits in some conditions and not others.

**Fig. 4:**
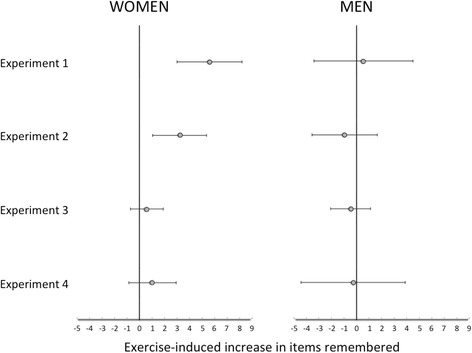
Means and 95% confidence intervals for each experiment indicating how many more paired associations were correctly recalled among female and male participants when the post-learning activity was exercise, relative to the non-exercise post-learning activity. For experiment 3, *error bars* reflect a repeated measures design, whereas those for the other experiments reflect independent measures designs. A meta-analysis across these experiments indicated that, among the female participants and with 95% confidence, 5 minutes of post-learning exercise increased memory for paired association by 0.40 to 4.63 items

In the present experiments, the evidence for retrograde, exercise-induced memory enhancements was consistent but noisy (see, for example, experiments 3 and 4, which replicated the pattern but not the statistical significance of the effect). The effects emerged among women but not among men, and the strength of the main effects varied across experiments. The somewhat noisy nature of these findings is consistent with the broader literature on the cognitive impact of acute exercise, where meta-analyses have concluded that the benefits are generally small and moderated by factors such as the temporal relationship between exercise and testing, the physical fitness of participants, the type of cognitive task, and exercise intensity (Chang et al., 2012). In a bid to accommodate such noise within the current set of experiments, a random-effects meta-analysis combined experiments 1–4, indicating that, with 95% confidence, the exercise-induced retrograde memory enhancement in women amounted to an increase of 0.40 to 4.63 paired associations that were correctly recalled (see Fig. 4 for means and 95% confidence intervals for each experiment). In contrast, an identical meta-analysis for the men indicated that, with 95% confidence, this enhancement (or lack thereof) amounted to an increase of −1.15 to 0.07 paired associations that were correctly recalled.^3^ A random-effects meta-analysis comparing the impact of exercise (versus non-exercise) among the women to the impact of exercise (versus non-exercise) among the men indicated that, with 95% confidence, the exercise-related increase in memory performance among women was 0.69 to 4.76 more paired associations than among the men.

As can be seen in Figs. 1 and 2, in the non-exercise control conditions female participants tended to perform worse than males—a difference that disappeared in the post-learning exercise condition. One not entirely satisfying possibility is that males did not exhibit exercise-induced retrograde benefits because they were already performing at near-ceiling levels. (On one hand, with a potential maximum of 15 items correct, performance might not appear to have approached ceiling, but it is possible that the males were performing more in line with the upper limits of their capacity given the experimental conditions.) Note that this possibility may help reconcile the apparent benefits of immediate exercise observed here with previous findings of such benefits only when exercise followed encoding by several hours (Van Dongen et al., 2016): in the previous study, it was hypothesized that memory was not enhanced by immediate exercise because performance was already optimized, which may be consistent with the current findings that benefits of immediate exercise were observed among the group that exhibited poorer memory under control conditions.

However, this leaves unanswered the question of why the female participants—as a group—performed worse than the males in the control conditions. In experiments 1 and 2, the stimuli were male faces and names, so it may be that the male participants were exhibiting an in-group benefit relative to female participants. However, although women have been found to remember female faces better than male faces, their memory for male faces has been found to be equivalent to that of male participants viewing the same stimuli (Lovén, Herlitz, & Rehnman, 2011). A potential in- versus out-group effect also would not account for the apparent retroactive benefits of post-learning exercise among women in experiment 3 (reflected in the modest but positive correlation between increase in heart rate and memory performance), unless one were to assign in-group/out-group status to the male names that were paired with abstract shapes in that study. The sex difference in the effect is also unlikely to be due to the exercise not effectively inducing physiological arousal within the male group, as in experiments 1 and 3 the male and female groups were statistically equivalent in the degree to which exercise increased heart rate. However, in both experiments, the female group showed slightly greater increase in heart rate; it may be that a more precise measure of physiological arousal—and of the accompanying release of stress hormones—would account for the sex difference we observed. Indeed, the literature on sex differences in physiological response to exercise is large and complex, with studies variously targeting different stress hormones and other physiological measures (e.g., Wideman et al., 1999); thus, it may yet be that the exercise manipulation in our study differentially impacted physiological mechanisms in men and women. Given previous findings that the time course of exercise-induced changes in stress hormones depends on the hormone measured (e.g., De Vries et al., 2000), it may also be that memorial benefits would have emerged more robustly and regardless of sex with a more intense and prolonged bout of exercise. It may in fact be that the sex differences revealed here stem from interactions between sex hormones and stress hormones that rise early during acute exercise, such as adrenaline and noradrenaline. Limitations of the current study include that we did not directly measure stress-related hormone levels and that we did not measure and account for individual differences in cardiovascular fitness.

An additional possibility that might account for the sex difference in our experiments is that stress hormones released through exercise interacted with menstruation-related hormones within a subset of the female participants. The administration of progesterone—a hormone that changes in concentration over the course of the menstrual cycle—has been found to impair memory, particularly for faces (Wingen et al., 2007), and it may be that post-learning exercise-induced arousal in the current studies exerted memorial benefits by counteracting such costs. Consistent with this possibility, a growing number of studies have documented sex differences and interactions between menstrual and stress hormones in the modulation of memory (e.g., Andreano et al., 2008; Andreano & Cahill, 2006; Felmingham et al., 2012; Nielsen et al., 2013). Unfortunately, we did not collect information about female participants’ menstrual stage or use of hormonal contraception, although this could be a worthwhile addition to future studies on this topic.

It is also important to note additional limitations in the current set of experiments. Although evidence for a memorial benefit from post-learning exercise emerged among female participants in experiments 1–3 (with a non-significant pattern in the same direction in experiment 4), the evidence in support of this conclusion took somewhat different forms in the different experiments. In experiments 1 and 2, the benefits robustly emerged in a comparison of means between the exercise and non-exercise conditions. In the third experiment, no such difference in means emerged but evidence of a modest correlation between exercise-induced change in heart rate and memory performance did. In contrast to the robust main effects in the first two experiments, this correlation fell short of statistical significance. As mentioned above, one possibility is that the memorial benefits of post-learning exercise are particularly apparent when trying to remember faces (instead of abstract shapes). Indeed, studies using emotion inductions have found that such manipulations induce retrograde memory enhancements for stimuli that were already particularly salient in participants’ minds (Sakaki, Fryer, & Mather, 2014); it may be that the retrograde impact of physical exercise is similarly constrained and that abstract shapes are simply less salient than faces. (Note that the relative salience of male faces for male and female participants might offer another explanation for the sex differences in our experiments. If so, then one prediction might be that the sex differences would reverse when the memoranda are composed of female faces.) Yet, the hint of a correlation in the predicted direction in experiment 3 suggests that the mechanisms underlying memorial benefits from post-learning exercise generalize to other types of stimuli. It may be that the incorporation of more precise indices of physiological arousal, such as peripheral levels of epinephrine and norepinephrine, could eliminate noise in the data by enabling more direct measurement of the hypothesized neurochemical basis of the effect than could be achieved through the fingertip pulse oximeter employed here. Similarly, studies following up on this work might benefit from more systematic manipulation of the intensity of exercise, as some measurement noise may have stemmed from variation in the gusto with which participants engaged the exercise task (Chang et al., 2012). Incorporation of a baseline memory measure—prior to the exercise versus non-exercise manipulation—might also have been one way to increase the precision with which we measured the memorial impact of the manipulation, as might have been measures of participants’ physical fitness (Chang et al., 2012). Given the ample opportunity for future experiments to improve upon the precision of measures in the current experiments, it is perhaps even more striking that the present patterns and conclusions were largely consistent across experiments.

## Conclusions

Across four experiments, participants who engaged in 5 minutes of low-impact exercise immediately after learning showed better recall for paired associations. This effect was consistently observable only among female participants for reasons that are not yet clear. Although the current experiments include both direct and conceptual replications of this pattern, it is not clear whether there are true sex differences in the mechanisms through which post-learning exercise enhances memory, or whether men would enjoy equivalent benefits under different conditions (e.g., when trying to remember female names and faces). Results also suggested that the memorial benefits of exercise-induced arousal reflect post-learning processes such as consolidation, as equivalent exercise prior to learning yielded no such benefits, although it may be that more variables must be measured in order to draw firm conclusions about the temporal relationship between memory and acute exercise. Additional work—as always—is necessary to resolve questions raised by the current set of experiments. Nevertheless, the present results suggest that, under some conditions, a short bout of exercise immediately after learning might aid in retention of learned material.

## Footnotes

1 One participant did not complete the self-reported mood measure.

2 Although there was a significant difference between the exercise and non-exercise conditions in self-reported mood measures of arousal, correlations between heart rate and memory performance were not substantially different when mood measures of arousal were partialed out.

3 Because meta-analytical methods are less equipped to accommodate structural dependence of data within repeated-measures designs (Peters & Mengersen, 2008), the repeated-measures experiment (experiment 3) was treated as between-subjects for the purpose of this meta-analysis.
